# Laser Polishing of Additive Manufactured Aluminium Parts by Modulated Laser Power

**DOI:** 10.3390/mi12111332

**Published:** 2021-10-30

**Authors:** Markus Hofele, André Roth, Jochen Schanz, Johannes Neuer, David K. Harrison, Anjali K. M. De Silva, Harald Riegel

**Affiliations:** 1 Laser Application Centre, Aalen University, Beethovenstraße 1, 73430 Aalen, Germany; andre.roth@hs-aalen.de (A.R.); jochen.schanz@hs-aalen.de (J.S.); johannes.neuer@hs-aalen.de (J.N.); harald.riegel@hs-aalen.de (H.R.); 2 School of Computing, Engineering and Built Environment, Glasgow Caledonian University, Cowcaddens Rd, Glasgow G4 0BA, UK; d.harrison@gcu.ac.uk (D.K.H.); A.DeSilva@gcu.ac.uk (A.K.M.D.S.)

**Keywords:** additive manufacturing, laser remelting, 3D printing, selective laser melting (SLM), surface quality, aluminium AlSi10Mg, laser powder bed fusion (L-PBF), surface remelting

## Abstract

In this study a new approach to laser polishing with periodic modulated laser power in the kilohertz regime is introduced. By varying the modulation frequency and modulation time, different periodic laser power curves with varying minimum, peak and average laser power can be created. The feasibility of the method is shown by polishing of vertical built AlSi10Mg L-PBF parts with an initial roughness of Ra = 12.22 µm. One polishing pass revealed a decreasing surface roughness with increasing energy density on the surface up to Ra = 0.145 µm. An increasing energy density results in a rising remelting depth between 50 and 255 µm and a rising relative porosity of 0.3% to 4.6%. Furthermore, the thermal process stability, analysed by the melt pool length in scanning direction, reveals a steadily increasing melt pool dimension due to component heating. Multiple laser polishing passes offers a further reduced surface roughness, especially at higher modulation frequencies and provides an improved orientation independent roughness homogeneity. The process stability regarding varying initial surface roughness revealed an almost constant relative roughness reduction rate with an achievable roughness variation after two polishing passes between Ra = 0.13–0.26 µm from an initial state of Ra = 8.0–19.2 µm.

## 1. Introduction

Laser powder bed fusion (L-PBF) is the most common additive manufacturing technique for rapid prototyping and industrial manufacturing of complex individual metal parts with small batches. The main advantages of this technology are a broad range of useable metals with the possibility to fabricate individual complex parts with good mechanical properties [1,2]. Based on the ability to fabricate parts directly from CAD models, geometric freedom increases and enables complex part geometries and shapes [3]. However, the layer-wise powder-based melting process causes a rough surface containing partly fused or sintered powder particles, which is inadequate for industrial applications, especially for cleanroom applications, food industry or medical implementation [4,5,6]. The emerging roughness depends on the shape of the individual part orientation and the position during the manufacturing process [3,7,8]. Possibilities to reduce the roughness in the LPBF process itself are limited [9,10,11].

**Figure 1:**
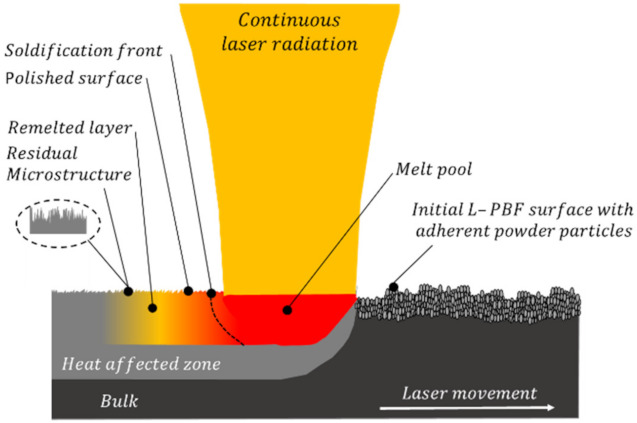
Schematic illustration of laser macro polishing with continuous laser radiation.

Laser polishing by surface remelting as a volume-maintaining technology can reduce the surface roughness and initial surface structures of metallic parts [12]. Laser polishing is a contact-free post processing technique which can handle complex freeform geometries [13]. During macro polishing a thin layer of the surface of the parts is molten by the laser beam, see Figure 1. Within the melt pool it results in a material flow from the peaks to valleys of the surface topography, driven by the surface tension and capillary forces and a heat-affected zone underneath [14]. In addition, adhering powder particles from the L-PBF process are melted into the surface. 

Laser polishing of additive manufactured metal parts is commonly done with pulsed or continuous laser radiation. Investigations on cobalt-chromium alloys (CoCr) [15,16,17,18,19,20], Inconel 718 [21,22,23,24], Inconel 625 [25], tool steel [26], tool steel H13 [27], tool steel S136H [28], maraging steel 1.2709 [29], Titanium Ti6Al-4V [18,30,31,32,33,34,35] and corrosion resistant steel 316L [36,37,38], 1.4542 [39] and LaserForm ST-100 [40,41] reveal roughness reduction rates between 50 and 90%. 

Aluminium alloys are frequently used for L-PBF. Due to the low absorptivity in the near infrared wavelength, which causes high back reflections and an unstable melt pool, laser surface processing of aluminium alloys is challenging and requires high beam intensities [42,43]. Furthermore, the laser surface treatment of aluminium is challenging due to high thermal conductivity, resulting in thermal losses and tends to cause rapid component heating, which can lead to process instabilities. 

Laser macro polishing of vacuum pressure die cast parts AlSi9MnMg with a solid state laser can reduce the arithmetic roughness Ra with pulsed laser radiation from 2.34 µm to 0.19 µm, and to Ra = 0.15 µm with continuous laser radiation, respectively [43]. Beam intensities at pulsed mode polishing of 850–1250 W/mm^2^ are found to be best.

Laser polishing of L-PBF-Aluminium AlSi10Mg parts with pulsed and continuous laser radiation can reach high roughness reduction rates up to 98% Ra [44,45,46,47,48]. At a pulse duration of 0.3 ms, a beam intensity of 1285 W/mm^2^ and an energy density of 76.5 J/mm^2^ the highest roughness improvement from Ra = 7.9 µm to Ra = 0.66 µm on vertical built samples is reached [45]. Multiple laser polishing with up to four scanning passes from different direction offers a further reduction to Ra = 0.14 µm at an area rate of 1 cm^2^/min. Micro polishing with a nanosecond laser system revealed the highest roughness improvement of 88% with a laser spot diameter of 85 µm, with an energy density of 12 J/cm^2^ and 15 scanning passes. Polishing with continuous laser radiation at a wavelength of 1.03 µm was found to be best with a laser beam intensity of 1057 W/mm^2^ and an energy density of 42 J/mm^2^. Thereby a roughness improvement of Ra = 0.23 µm with single polishing, and 0.14 µm with four times polishing at an area rate of 5 cm^2^/min is reached [45]. Polishing with a CO2 laser (10.6 µm) the highest roughness improvement of 85% from Sa = 22.3 µm to 7.9 µm with the highest tested energy density of 330 J/mm^2^ is reached [46]. Pulsed and continuous mode laser polishing of L-PBF surfaces with varying initial roughness by changing fabrication angle revealed a constant relative roughness reduction ability [49]. With decreasing the fabrication angle and thus increasing the initial roughness, the partial roughness regarding to residual long periodic surface structure is increased. Material analysis of the remelting zone, polished with a solid state laser (1.03 µm) at continuous laser radiation, revealed an increasing remelting depth from 50 µm up to approximately 150 µm with rising beam from 0.9 kW/mm^2^ up to 1.6 kW/mm^2^ [48]. With pulsed mode polishing the remelting depth is almost constant in the range of 130 µm to 160 µm. The porosity of the remelting zone varies between 0.98% and 1.7%. The micro hardness, within the molten surface layer, varies from 105 to 96 HV 0.1 [48]. The analysis of the residual laser polished surface structure and the chemical composition by SEM and EDX revealed an increased grain size and an extended amount of silicon and magnesium. Bright particles on the polished surface could be detected as aluminium oxides [48]. 

Surface remelting with modulated laser radiation, used for surface structuring and the creation of design surfaces on Inconel 718 and hot work steel H11 by material relocation with peak to peak distances several times larger than the laser spot diameter and modulation frequencies of less than 200 Hz could be achieved [50,51]. On H11 periodic surface profiles with wavelengths between 0.25 and 4 mm and structure heights up to 12 µm can be created [51]. 

This paper introduces a new approach to laser polishing by modulated laser power. Hereto a process parameter investigation on laser polishing of additive manufactured Aluminum AlSi10Mg parts with a laser power modulation in the kilohertz regime is used in order to combine high peak powers for a stable energy in-coupling with high average laser powers and area rates. Hereto the modulation behaviour at modulation frequencies between 2 kHz and 5 kHz of the used laser system is characterised. Laser polishing with varying minimum, peak and average laser power is performed on vertically built rectangle samples. The resulting surface roughness and topography are analysed by means of microscopy, 3D profilometer and scanning electron microscope SEM. The remelting depth and porosity of the remolten surface layer depending on the energy input is examined by cross sections. The thermal stability and melt pool size are analysed by means of high speed camera images. Additionally, roughness improvement by varying the number of polishing passes and the scanning direction is investigated. The process stability and roughness reduction ability at varying initial roughness, caused by different fabrication angles, are analysed and compared to the common continuous and pulsed laser operation modes. 

## 2. Experimental Design

### 2.1. Applied Laser Polishing Setup

**Figure 2:**
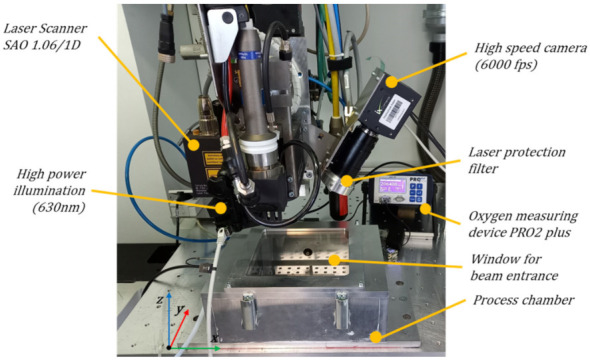
Experimental setup at the TRUMPF Laser Cell TLC 40 with a SAO 1.06/1D scanner optics, process chamber, oxygen measurement device PRO2 plus and high-speed camera I-Speed 221 with illumination (630 nm).

Laser polishing was carried out by means of a disk laser of the type TruDisk 4002 (TRUMPF, Schramberg, Germany) with a maximum output power of 4000 W. The laser offers the possibility to modulate the laser power in the kilohertz regime from 1–5 kHz with modulation times (laser pump module active time) from 100 µs up to continuously. The laser beam was guided with a 200 µm gradient index fibre with a Numerical Aperture NA of 0.1 to a 5-axis Trumpf Laser Cell TLC 40 (TRUMPF, Ditzingen, Germany), Figure 2. 

The laser polishing took place in a purified inert gas atmosphere within a sealed process chamber. The residual oxygen concentration was controlled during the laser polishing process with an oxygen-measuring device of type PRO2 plus (Orbitalservice, Heimbuchenthal, Germany). The melt pool formation is observed by use of a high-speed camera of type I-Speed 221 (IX cameras, Rochford Essex, United Kingdom) with a frame rate of 6 kHz. The illumination of the process zone is done by means of a high power LED module with a wavelength of 630 nm. 

The beam deflection is carried out by means of a scanner optics of type SAO 1.06/1D (Frauenhofer IWS, Dresden, Germany) with a focal length of 230 µm resulting in a focal diameter of 450 µm, Figure 2. The scanner system enables a one-dimensional (1D) laser beam deflection with a maximum frequency $f_{p}$ of 300 Hz in combination with a maximum pendulum length of 70 mm. The controller of the scanner offers the ability to adjust the laser power by sectioning the pendulum movement into 15 segments, where the laser power of the pump modules can be freely adjusted by the operator. The average pendulum speed $v_{p,avg}$ over the work piece surface is given by the pendulum frequency $f_{P}$ and the pendulum width *x*, see Equation (1).

(1) $$v_{P,avg}=2\cdot x\cdot f_{P}$$

**Figure 3:**
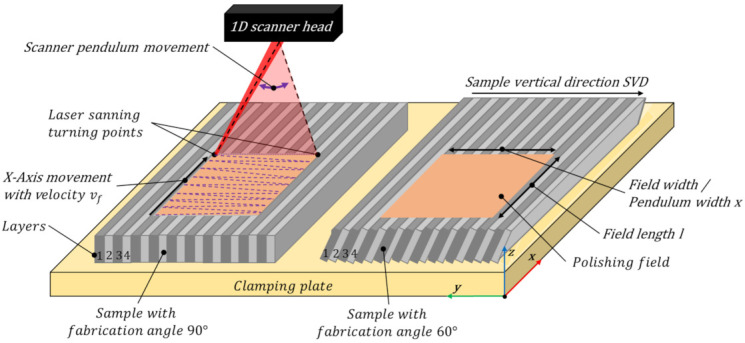
Schematic description of the beam guidance by means of the 1D scanner and superimposed axis movement. Sample orientation on the clamping plate in the process chamber during laser polishing, left: 90° degree fabrication angle, right: 60° degree fabrication angle.

In Figure 3, a schematic image of the pendulum movement, using the scanner optics, in conjunction with the machine axis is given. At laser polishing, the aluminium specimens were mounted horizontally on a clamping plate. Thus, the y-direction is equal to the sample vertical direction (SVD) and the laser beam is steadily vertically orientated according to the sample surface in z-direction over all samples, and over all fabrication angles, respectively.

The pendulum movement in y-direction is superimposed by the x-axis movement of the scanner head with the velocity $v_{f}$. The resulting average beam velocity $v_{l}$ is calculated by the following Equation (2):

(2) $$v_{l}=\sqrt{v_{p,avg}^{2}+v_{f}^{2}\;}$$

The revealing area rate AR of laser polishing is calculated from the axis velocity $v_{f}$ and the pendulum width x, according to Equation (3).

(3) $$AR=v_{f}\cdot x$$

The resulting energy input per unit area, the energy density ED, is calculated by the following Equation (4). For an areal treatment, *ED* is given by the mean laser power $P_{l}$, the pendulum width *x* and the axis velocity $v_{f}$.

(4) $$ED=\frac{P_{l}}{x\cdot v_{f}}$$

The average percentage track overlap $TO_{av}$, measured at the center of the polishing field, is given by the axis velocity $v_{f}$, the pendulum frequency $f_{p}$ and the beam diameter at the work piece surface $d_{L}\left(z\right)$, see Equation (6).

(5) $$PPO_{av}=\left(1-\frac{\left[v_{l}\cdot \left(\frac{1}{f_{mod}}\right)\right]\;}{d_{L\;}\left(z\right)}\right)\cdot 100\%$$

The average percentage track overlap $TO_{av}$, measured at the centre of the polishing field, is given by the axis velocity $v_{f}$, the pendulum frequency $f_{p}$ and the beam diameter at the work piece surface $d_{L}\left(z\right)$, see Equation (6).

(6) $$TO_{av}=\left(1-\frac{v_{f}}{f_{p}\cdot d_{L\;}\left(z\right)}\right)\cdot 100\%$$

### 2.2. Measurement Devices and Evaluation Methods

The initial and polished surfaces are analysed quantitatively and qualitatively. The quantitative analysis of the surfaces roughness, i.e., Ra and Rz, according to EN ISO 4288:1997, was realised tactilely by means of a perthometer of type MarSurf M400 (Mahr, Göttingen, Germany). According to standard ISO 4288 for the initial surface with Ra > 2 μm, a cut-off wavelength of 2500 μm was used, whereas for the polished surfaces with Ra < 2 μm the cut-off wavelength is determined to 800 μm. The shown roughness values are mean values based on ten measurements from the initial surfaces, five measurements per polishing field and measuring direction, respectively. Therefore, the positions of the individual measurements were distributed homogeneously over the polishing field and the test plates, respectively. 

The surface is further analysed by a Fourier transformation of the measured tactile surface profiles. Hereto the tilt of the sample is deducted in the measurement data and levelled to zero. The result of the Fourier transformation is clustered into a logarithmic scale of the spatial wavelength. For each range of the spatial wavelength the partial roughness Ra is calculated.

**Figure 4:**
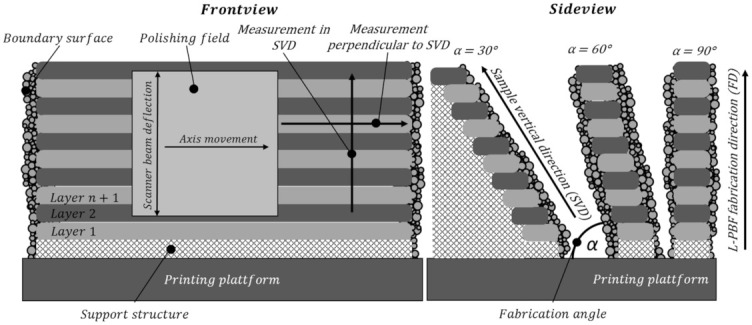
Definition of the L-PBF fabrication, polishing and measurement directions on the AM samples.

In this paper, polishing and measuring directions were varied. In Figure 4, a schematic image of the layer-wised L-PBF samples with different fabrication, polishing and measurement directions is depicted. The initial surface topography and roughness were measured in the sample vertical direction (SVD) and perpendicular to SVD of the L-PBF process. In the SVD, the roughness structures caused by the layer wise production technology are taken into account. 

Laser polishing was basically done with the fast laser beam deflection of the scanner optics in the fabrication direction and the superimposed axis movement perpendicular to the fabrication direction, as shown in Figure 3. A single polishing movement perpendicular to the SVD is defined as: 1 × 0, and one polishing pass in the SVD as: 0 × 1. If laser polishing is repeated with two bidirectional passes perpendicular to SVD: 2 × 0 or a combination of one pass in SVD and one pass across SVD:1 × 1. Two repeated passing’s in both directions is defined as 2 × 2. After laser polishing, the surfaces were measured in the SVD and perpendicular to SVD, Figure 4. 

Qualitative evaluation of the 3D surface topography is carried out with a 3D profilometer VR-3100 from Keyence, Neu-Isenburg, Germany. The microstructure of the surface was analysed using an optical microscope of type Axio Zoom V16 (Carl Zeiss, Jena, Germany) with a 100-fold magnification and a scanning electron microscope (SEM) of type Sigma 300VP (Carl Zeiss, Jena, Germany). The remelting zone depth, relative porosity and pore size distribution are analysed on polished and etched cross sections in x-direction with a 200-fold magnification by use of a Carl Zeiss Vario Axio Imager.Z2 Vario. The porosity detection is based on automatic grey scale edge detection.

### 2.3. Material and Samples

The experimental investigations are executed on the Aluminium alloy AlSi10Mg. Rectangular plates with the dimension of 100 mm length, 30 mm width and a material thickness of 3 mm were built up by laser powder bed fusion (L-PBF) on a SLM280HL from SLM Solutions, Lübeck Germany. The machine has a fabrication chamber size of 280 × 280 × 280 mm^3^ and a 400 W Yb-fibre laser. The used aluminium powder has a powder grain diameter D10 of 26.4 µm and D90 of 71.0 µm. The average powder grain diameter D50 amounts to 43.1 µm. The samples were built with recommended fabrication parameters, provided by the SLM Solutions, Lübeck, Germany, with a slicing thickness of 50 µm. The layers are melted on the outer contour with a laser power of 350 W and a beam velocity of 600 mm/s. The core of the part is exposed with 350 W, 1150 mm/s and a hatch distance of 170 µm, respectively. In order to investigate the influence of different fabrication orientations and their dependency on the initial roughness after 3D printing and the achievable roughness after laser polishing, the samples are printed in different fabrication orientations. Therefore, the fabrication angle is varied from 15° to 90° in steps of 15°. Samples with 45° fabrication angle and less are built up with full support structures at the overhanging backside surface, see Figure 4. 

After cutting the samples from the L-PBF built platform the initial surface of the 3D printed parts was analysed. Before laser polishing, the parts are pre-treated by laser cleaning in order to ablate and reduce the oxide layer and the residual powder particles from 3D printing. The laser cleaning process was realised with a short pulse laser TruMark 5020 (TRUMPF, Schramberg, Germany) with an average laser power of 20 W at a pulse duration of 70 ns and a pulse frequency of 65.5 kHz. The beam with a diameter of 122 µm was guided three times over the surface with a track offset of 70 µm and a scanning velocity of 3000 mm/s.

For an examination of the initial surface roughness, the surfaces of the front and back side of the samples is measured according to Figure 4 in sample vertical direction (SVD) of the L-PBF process and perpendicular to the SVD (along the layers). The measured mean surface roughness Ra and the mean roughness depth Rz in both directions for the used vertical printed parameter investigation specimens are given in Table 1.

**Figure 5:**
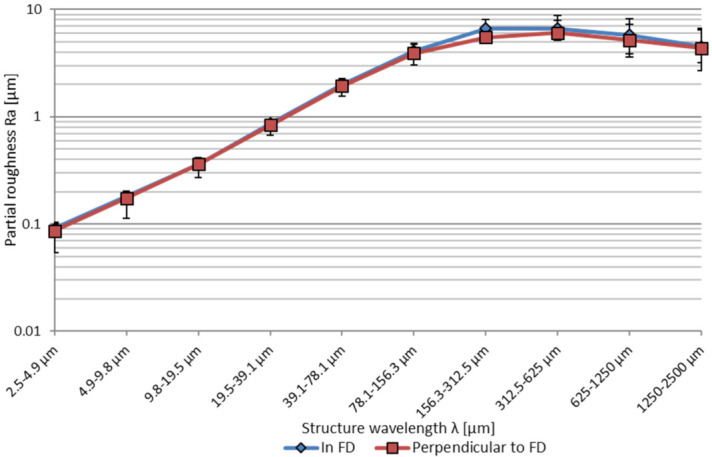
Partial roughness Ra of the vertical fabricated samples (fabrication angle 90°), used for the process parameter investigations, depending on the measuring direction.

When considering Ra and Rz, the surface roughness perpendicular to SVD is slightly lower compared to in sample vertical direction, which is in this case equal to the fabrication direction. The partial arithmetic roughness, analysed by means of a Fourier transformation of the roughness profiles shows that the medium and long wavelength roughness structures larger than λ = 40 µm represent the dominant part of the surface roughness, see Figure 5. Between the orientations, only comparatively small differences in the spatial roughness greater than λ = 156 µm can be observed. In this region the partial roughness in fabrication direction (FD) from λ = 156 µm–1250 µm is higher than perpendicular to FD. 

**Figure 6:**
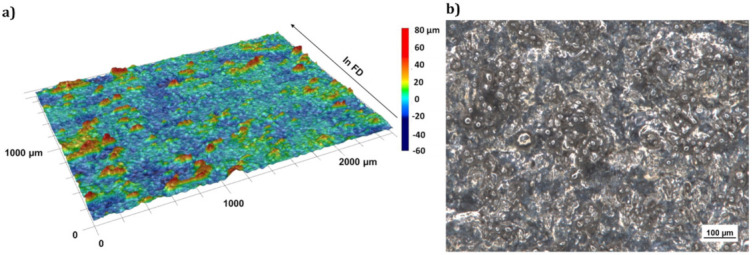
(**a**) 3D topography of the initial surface structures with 12-fold magnification, (**b**) 100-fold microscopic image of the initial surface.

Figure 6a shows the 3D surface topography of the used parts, measured at the middle of the sample surface. The surface exhibits a global waviness, which is superimposed with particular protruding material accumulations (red coloured areas Figure 6a), which are mostly elongated and perpendicular to FD. In more detail, under a 100-fold microscopic image it gets visible that the surface consists of a superior structure where a lot of powder particles are stick on (Figure 6b).

In order to analyse the influence of the initial surface roughness on the achievable surface roughness, specimens with varying fabrication angle and identic geometric dimensions are created on a second printing job. Parts with fabrication angles below 45° degrees are fully supported on the overhanging backside. With increasing fabrication angle the surface roughness is decreasing at the frontside and backside. At the frontside surface the roughness is decreasing from 18.6 µm to 8.2 µm, Table 2. The overhanging backside of the parts at a 45° degree fabrication angle exhibits the highest arithmetic surface roughness Ra of 19.2 µm, which is almost double as high compared to the frontside of the specimen. 

## 3. Results and Discussion

### 3.1. Characterisation of Laser Modulation Behaviour

**Figure 7:**
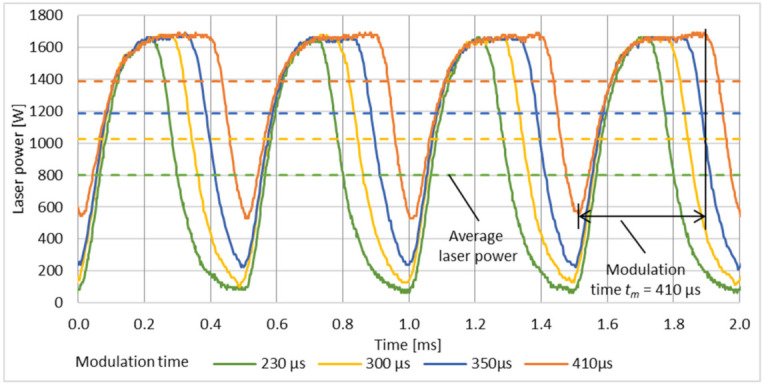
Modulation behaviour at varying modulation time *t_m_* at a modulation frequency *f_m_* of 2 kHz.

Laser power modulation of continuous wave laser systems is done by means of the modulation of the pump modules of the resonator. The laser power modulation behaviour of the laser system depends on design and characteristic of the resonator. It influences the rise time and decay time of the laser intensity in the resonator. The solid state disc laser system TruDisk 4002 offers modulation frequencies *f_m_* between 1 kHz and 5 kHz at variable modulation times *t_m_*. The modulation time, period of time marked, in Figure 7, represent the pump module on time, respectively, and the time in which energy is applied to the resonator via the pump modules at the set laser power. In order to analyse the modulation behaviour, the decoupled laser emission from the resonator is measured with the internal laser power sensor (called LEM) and recorded by use of an oscilloscope. 

**Figure 8:**
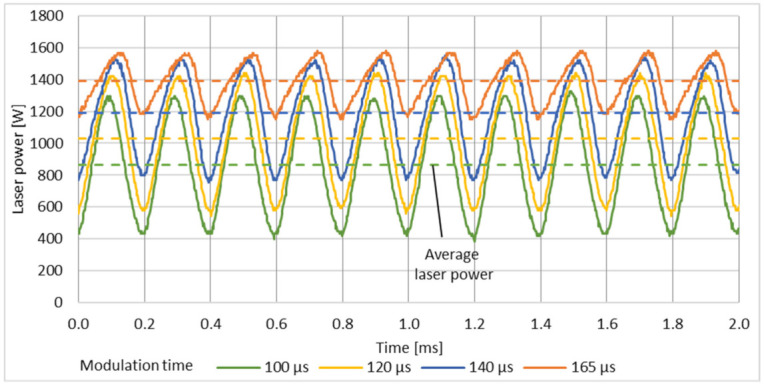
Modulation behaviour at varying modulation time *t_m_* at a modulation frequency *f_m_* of 5 kHz.

The laser power of the modulated laser emission was fixed to 1700 W, which was found to be suitable for pulsed mode laser polishing on additive manufactured L-PBF aluminium surfaces [45]. The resulting peak power, average laser power and constant minimum laser power of the modulated laser emission are adapted, by adjusting the modulation time, see Figure 7 and Figure 8. At a modulation frequency *f_m_* of 2 kHz and a modulation time between 230 µs and 410 µs, the peak power is almost constant with 1680 W–1712 W. The minimum laser power varies from 60 W to 528 W. The average laser power increases from 801 W at *t_m_* = 230 µs to 1387 W at *t_m_* = 410 µs. 

At the laser specific maximum modulation frequency *f_m_* = 5 kHz the peak power is no more constant, see Figure 8. With increasing modulation time *t_m_* from 100 µs to 165 µs the peak laser power increases from 1296 W to 1568 W. The set laser power of 1700 W is not reached due to too short pump times. Short pump module off times at high modulation frequencies in combination with the given decay time of the laser radiation in the resonator causes a significant rise in the minimum laser power. Thus, the minimum laser power is in the range of 416 W–1168 W. With increasing the modulation time, the laser power emission gets more and more to an emission behaviour comparable to continuous operation mode with less difference in base and peak load.

The complete measured laser power emission parameters, which were used for the further laser polishing investigations can be taken from Table 3.

### 3.2. Constant Process Pararameters during Laser Polsihing

The process investigations were applied on a test field matrix with field dimensions of 10 × 10 mm^2^. The fields had a distance of 2 mm between each other, and the edge distance was at least 3 mm. A latency time of 30 s between polishing of two fields was considered to prevent a significant rise in temperature of the aluminium substrate. The investigations with modulated laser power emission are executed with a defocused laser beam diameter *d_s_* of 1298 µm (focal position 12 mm), which was measured by a focus monitor system FM+ from Primes and a constant peak to peak overlap *PPOav* and track overlap *TO*, which were found to be suitable for pulsed mode laser polishing at additive manufactured AlSi10Mg [45]. Detailed constant process parameters can be taken from Table 4.

**Figure 9:**
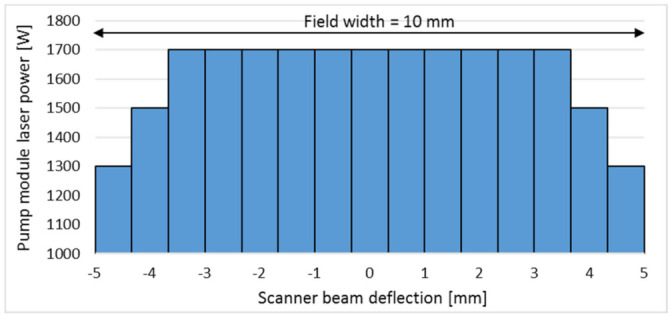
Adjustment of the pump module laser power over the scanner beam deflection (scanner pendulum movement).

Due to the 1D pendulum movement, two turning points emerge, where the laser beam velocity in y-direction is momentarily zero, which results in a constant laser power in a significant increased energy deposition on the turning points and the border of the polishing fields, respectively. To prevent this, the pendulum axis is sectioned into 15 segments with adjustable laser power. Therefore, a progressive reduction of the constant pump module laser power *P_L_* of 1700 W in two steps and of 200 W at both turning points is defined, which was found to be suitable for laser polishing with pulsed and continuous laser radiation in [45], see Figure 9.

### 3.3. Basic Process Parameter Investigation with Modulated Laser Power

The basic process parameters investigations on laser polishing with modulated laser emission are performed with a modulation frequency *f_m_* from 2 kHz to 5 kHz in steps of 1 kHz. In order to keep the peak to peak overlap *PPOav* and the track overlap *TO* constant, the pendulum frequency and axis velocity are adjusted depending on the modulation frequency *f_m_*. Consequently, the reached area rate is in the range of 10 to 25 cm^2^/min. The resulting energy density *E* varies between 20.8 J/mm^2^ and 834.2 J/mm^2^. The full experimental plan is given in Table 5.

#### 3.3.1. Achievable Surface Roughness Depending on the Modulation Behaviour and Energy Density

**Figure 10:**
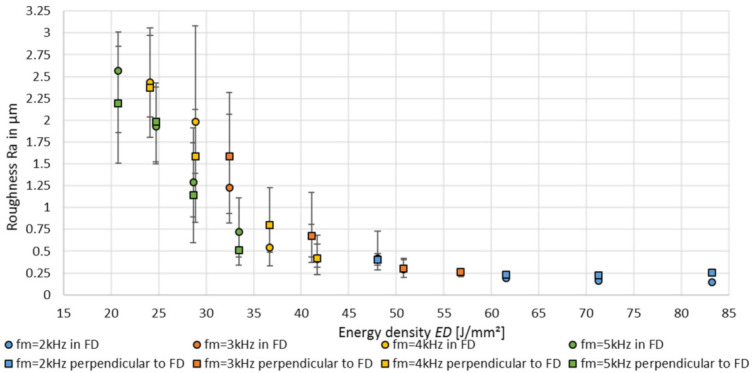
Achievable roughness Ra, depending on the modulation frequency, as a function of the energy density *ED*.

When considering the achieved arithmetic roughness Ra as a function of the energy density *ED*, an increase of the energy density *ED* correlates with a smaller roughness as well as a decreasing roughness variance, given by the scattering bars, see Figure 10. Within a modulation frequency *f_m_*, a significant roughness improvement with an increasing modulation time *t_m_*, which results in an increasing average laser power and energy density *ED*, is visible for both the measuring directions. The lowest surface roughness is achieved with the lowest modulation frequency *f_m_* of 2 kHz, which results in the highest energy densities, too. Thus, the minimum roughness of Ra = 0.145 µm was obtained with an energy density of *ED* = 83.2 J/mm^2^, measured in SVD, and perpendicular to SVD with Ra = 0.225 µm and an energy density of *ED* = 71.3 J/mm^2^, respectively. With energy densities *ED* below 45 J/mm^2^, higher modulation frequencies with a periodic laser power curve with a higher minimum laser power lead to a decreasing roughness at comparable energy densities in both the measuring directions, e.g., with *f_m_* = 4 kHz, *ED* = 28.9 J/mm^2^, Ra _in SVD_ = 1.98 µm versus *f_m_* = 5 kHz, *ED* = 28.6 J/mm^2^, Ra _in SVD_ = 1.29 µm. Further, with energy densities below 45 J/mm^2^ the surface roughness has a high orientation dependency, shown by the roughness variance between both the measuring directions. 

**Figure 11:**
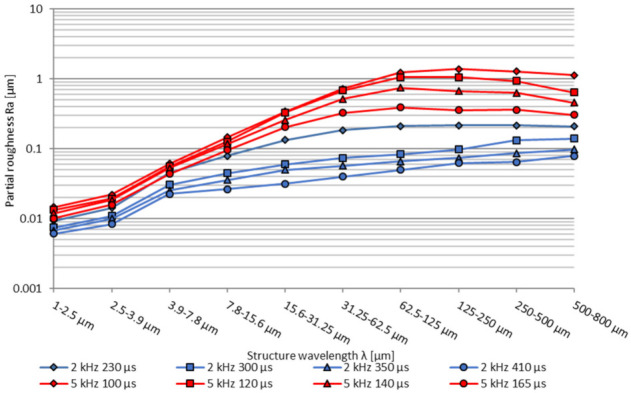
Partial roughness Ra, polished with modulation frequency *f_m_* = 2 kHz and 5 kHz depending on the modulation time *t_m_*, measured in sample vertical direction (SVD).

Looking at the partial arithmetic roughness, the lowest roughness Ra is achieved with the longest modulation time *t_m_* over the complete wavelength spectrum, see Figure 11. In general, an increase of the modulation time *t_m_* and energy density *ED* results in a smaller surface roughness. In addition, at a lower modulation frequency *f_m_* = 2 kHz, a significantly lower roughness is shown over the total structural wavelength range, compared to a modulation frequency of *f_m_* = 5 kHz. In the medium- and long wavelength structural range, larger differences between the individual modulation times can be seen for a modulation frequency of *f_m_* = 5 kHz compared to *f_m_* = 2 kHz. Overall, a significant roughness improvement in the medium and long surface structures is reached with increasing energy input *ED*, while short structure wavelength below λ = 7.8 µm exhibits only comparable small improvements, which is similar to pulsed and continuous mode polishing [45].

#### 3.3.2. Surface Appearance and Topography

**Figure 12:**
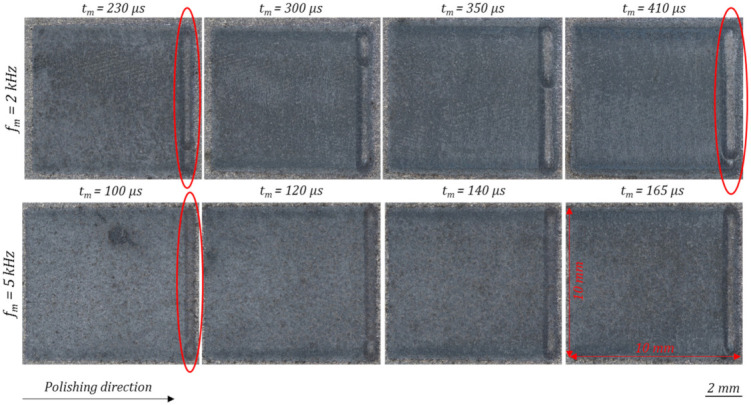
Microscopic pictures of the laser polished surface with a 25-fold magnification considering the used modulation frequencies *f_m_* and the modulation time’s *t_m_*.

In comparison to the laser-cleaned initial surface, which is visible at the borders of the microscopic images of Figure 12, the polished area is getting significantly darker. When comparing the polished areas, depending on the used process parameters (modulation frequencies *f_m_* and modulation times *t_m_*), minor differences in the appearances can be observed, see Figure 12. With increasing modulation time *t_m_* the surface appearance gets slightly darker. Similar darker areas were analysed in [48] by means of an EDX-analysis. Thereby, an increased oxygen content was measured. When comparing the widths of the last scanning tracks of each polishing field, framed in red colour, it can be seen that the melt pool width is increasing with rising energy input and modulation time, see e.g., Figure 12, *f_m_* = 2 kHz: *t_m_* = 230 µs vs. *t_m_* = 410 µs.

**Figure 13:**
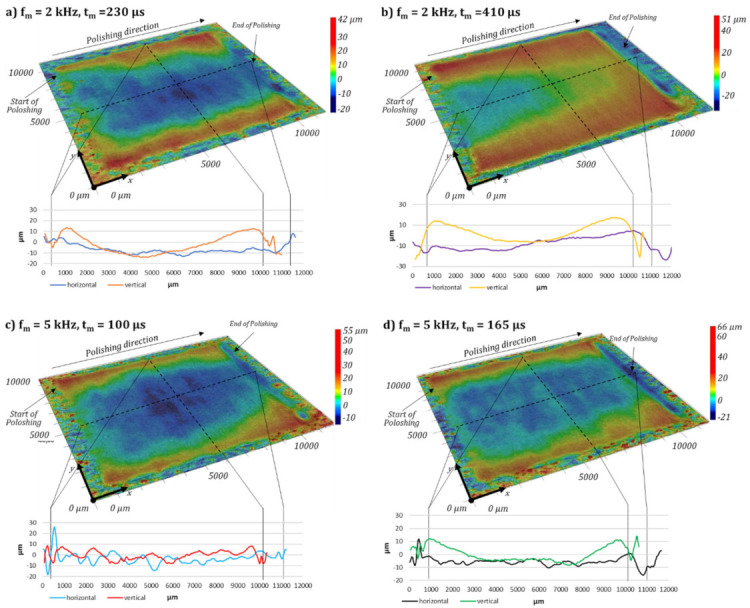
Fold magnification of the 3D topography of the laser polished surface structures with a modulation frequency of *f_m_* = 2 kHz (**a**,**b**) and *f_m_* = 5 kHz (**c**,**d**) depending on the modulation time *t_m_*.

A qualitative analysis of the achieved roughness after laser polishing, by 3D topography measurement, gives an impression of what causes the main differences of the surface roughness, see Figure 13. It should be noted that the height scale differs between the images. When comparing the initial L-PBF surface around the polishing field with the surface topography at the polished area, the initial short and medium surface structures, are flattened. The long-wavelength structures and height differences are still present. Hence, after laser polishing, a waviness with structure wavelengths above the cutoff wavelength of the roughness spectrum is still visible.

In general, laser polishing results in material transport from the centre of the polishing field to the upper and lower turning points of the scanner pendulum movement, similar to pulsed mode polishing [49]. Local immersion of up to −20 µm occur in the centre of the polishing field, Figure 13a. The material displacements can be taken from the height profile of the horizontal and vertical lines. At a modulation frequency of *f_m_* = 2 kHz the increase of the modulation time from *t_m_* = 230 µs to *t_m_* = 410 µs significantly increases the material accumulation with progressing polishing process in polishing direction. Compared to a modulation frequency of *f_m_* = 2 kHz, the material transport to the upper and lower end of the polishing field is reduced but still presents a modulation frequency of *f_m_* = 5 kHz, see Figure 13c,d. The surface topography remains approximately unchanged also with increasing the modulation time from *t_m_* = 100 µs to 165 µs, see Figure 13c,d. At the end of the polishing area along the last hatching track, depressions above 20 μm are obtained, which are also clearly visible in the horizontally oriented profile lines, e.g., Figure 13d).

#### 3.3.3. Influence of the Energy Density on the Remelting Depth and the Surface Structures

**Figure 14:**
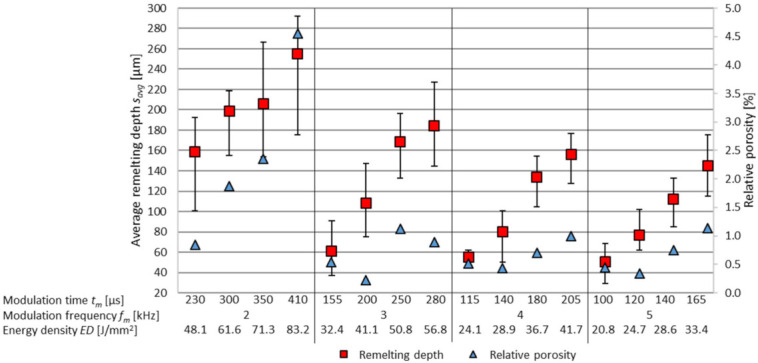
Average remelting depth *s_avg_* and relative porosity depending on the modulation frequency *f_m_* and modulation time *t_m_*.

The influence of the set modulation time *t_m_* and the selected modulation frequency *f_m_* on the resulting average remelting depth *s_avg_* is analysed by cross sections perpendicular to SVD through the centre of the polishing fields, see Figure 16. Additionally, the relative porosity, defined as pore area/total area of remelting, is evaluated. The relative porosity within this study varies from 0.3% to 4.6%, see Figure 14. At all modulation frequencies, an increase in the remelting depth *s_avg_* results in a simultaneous increase of the used energy density *ED*. The highest arithmetic remelting depth *s_avg_* of 255 µm is achieved with a modulation frequency of *f_m_* = 2 kHz and an energy density of *ED* = 83.2 J/mm^2^. At a remelting depth of *s_avg_* = 255 µm, a significantly higher relative porosity of 4.6% can be observed. With the exception of *f_m_* = 3 kHz the relative porosity increases considerably with an ascending energy density *ED* > 24 J/mm^2^, which correlates to the investigations on polishing with pulsed laser radiation, where the relative porosity tends to increase with increasing laser beam intensity [48]. 

Taking the energy density *ED* into account it becomes visible that with an increasing *ED* a higher remelting depth at each modulation frequency can be achieved. For example, at a modulation frequency of *f_m_* = 3 kHz, an average remelting depth of *s_avg_* = 60.8 µm is achieved with an energy density of *ED* = 32 J/mm^2^. If the *ED* is increased by 75% to *ED* = 56.8 J/mm^2^, the resulting remelting depth will increase by three-fold to 184.6 µm. 

**Figure 15:**
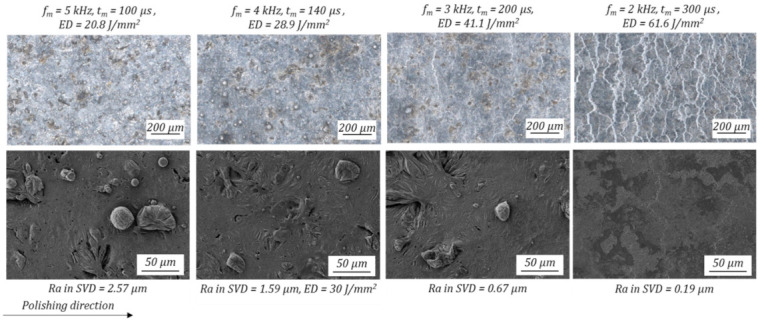
Microscopic differences of the achieved surface behaviour after laser polishing, depending on the energy density ED at the centre of the polishing fields. First row light microscopic images with a 100-fold magnification, second row SEM images with a 500-fold magnification.

The visual observation of the resulting surface behaviour after laser polishing is analysed by means of light microscope and scanning electron microscope, shown in Figure 15. At lower energy density, *ED* = 20.8 J/mm^2^ with Ra_inSVD_ = 2.57 µm, an incomplete melting of the initial surface topography occurs. Thus, the surface still consists of partly molten surface structures with λ > 100 µm, adhering spherical particles and linear surface depressions or defects. The dimension of the spherical particles ranges between 10 and 45 µm, which correlates mostly to the powder particle size distribution D10–D50 of 26.4–43.1 µm. With an energy density of *E*D = 28.9 J/mm^2^, which results in arithmetic roughness of Ra_inSVD_ = 1.59 µm, the waviness of long surface structures is reduced, but a high amount of adhering particles are still on the surface. Above *ED* = 40 J/mm^2^ residual surface defects decrease and only sporadically adhering powder particles can be detected, leading to a significant roughness reduction to Ra_inSVD_ = 0.67 µm. The microscopic image shows presence of bright linear surface feature. At Ra_inSVD_ = 0.19 µm, which was achieved with *ED* = 61.6 J/mm^2^, a strong increase of those linear surface feature can be detected on the microscopic image. When observing the SEM image, no residual structures can be detected. Thus, those bright linear surface features have no structure in the third dimension and an impact on the surface roughness. 

#### 3.3.4. Formation of Pores and Pore Size Distribution as a Function of the Process Parameters

**Figure 16:**
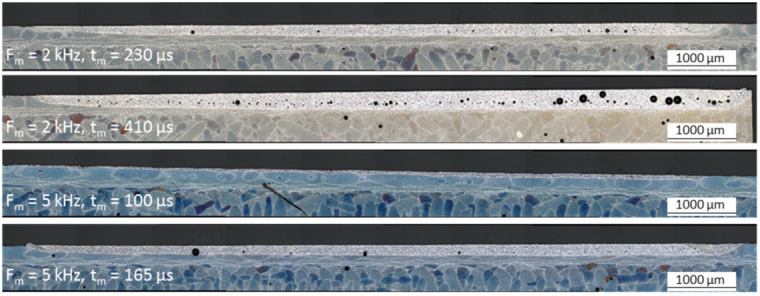
Etched cross sections of the remelting zone in direction of the axis movement.

Variation in the modulation frequency and modulation time, and the energy density *ED*, highly affect the relative porosity, as shown in Figure 14, and also has a major influence on the pore size distribution, see cross sections on Figure 16. Overall, the pore size is highly varying. Thus, within this study, the pore size at the remelting zone is in the range of 0.21–11,974.7 µm^2^. With increasing energy density *ED* an increase of large pores can be observed. Additionally, the large share of the pores are located in the deeper area of the remelting zone. 

**Figure 17:**
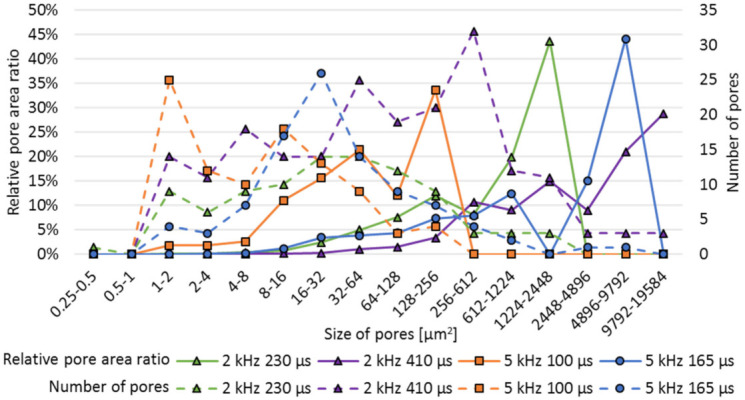
Pore size distribution depending on the modulation frequency *f_m_* and modulation time *t_m_*.

The pore size distribution within the remelting zone, given by the relative pore area ration, reveals a shift from a lot of small sized pores to several large pores with increasing modulation time, see Figure 17. With *f_m_* = 5 kHz, *t_m_* = 100 µs, the pore size varies between 0.5 µm and 211 µm^2^, but already 35% of the relative pore area is between 1 and 2 µm^2^. At *f_m_* =5 kHz, *t_m_* = 165 µs and the pore size is in the range of 1.4 µm–7425 µm^2^ and almost 45% of the relative pore area results from one pore with a size of 7425 µm^2^.

**Figure 18:**
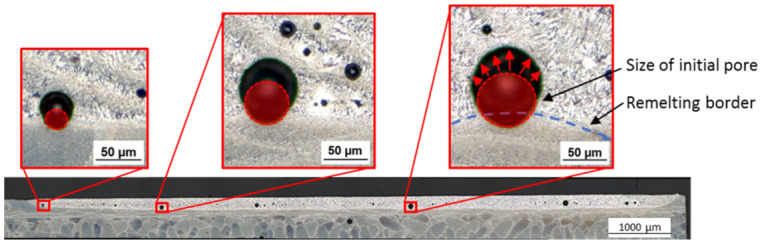
Expansion of pores from the L-PBF process at the border of the remelting zone.

The formation of pores within the remelting zone has to be differentiated into initial pores from the L-PBF process and process pores, like gas porosity from the polishing process. Several medium and large-sized pores, which were found in the remelted zone, are located at the border of the remelting zone and are partially in the unmolten zone below the remelting zone of the polishing process, see Figure 18. The area of the pores seems to extend by remelting this surface layer, when calculating the dimension of a circular initial L-PBF pore by using several measuring points at the unmolten bottom of the pores. When comparing the calculated red dotted circle at the microscopic images of Figure 18, a growth of the pore in the remolten area in surface direction can be assumed.

**Figure 19:**
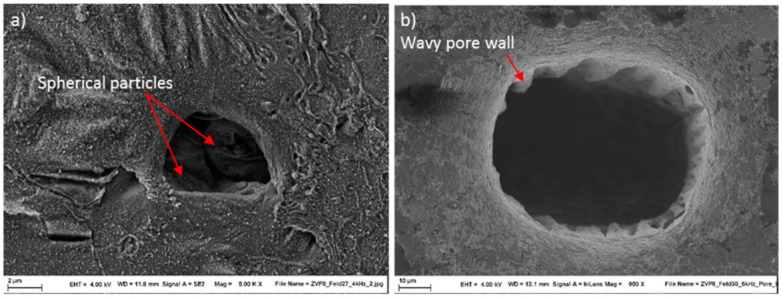
Surface open pores, (**a**) powder particles at the bottom, (**b**) deep pore with a wavy wall.

Additionally, some surface open pores can be detected. These pores are deep and exhibit partly a wavy wall of the pores (see Figure 19b). At the bottom of some pores spherical particles can be detected which may result from an initial defect in the part by lack of fusion within the L-PBF process (see Figure 19a).

### 3.4. Multiple Polishing Passes

Laser polishing with one pass achieved the highest roughness reduction at each modulation frequency at the highest energy input and modulation time, respectively. Hence, laser polishing with multiple passes is investigated with the highest modulation times. Between the polishing passes a latency time of 30 s is used in order to reduce the influence by global heating of the specimens.

**Figure 20:**
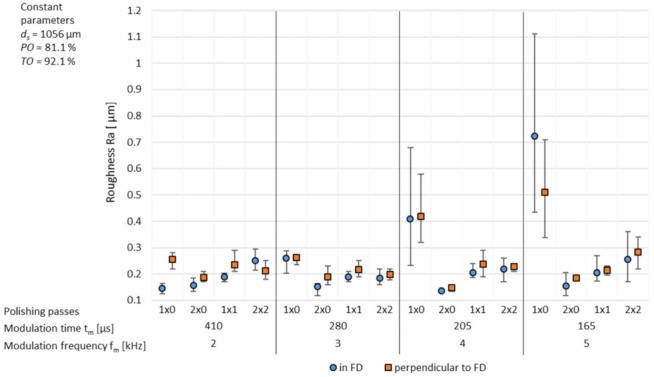
Achievable roughness Ra with multiple polishing passes depending on the modulation time *t_m_* and modulation frequency *f_m_*.

Looking at the roughness Ra in dependence of the number of polishing passes, the modulation time *t_m_* as well as the used modulation frequency *f_m_* the lowest roughness can be achieved with 1 × 0 passes using a modulation frequency *f_m_* of 2 kHz respectively, with 2 × 0 passes using a modulation frequency *f_m_* of 3/4/5 kHz, see Figure 20. While at a modulation frequency *f_m_* of 2 and 3 kHz, the achievable roughness Ra is relatively stable as a function of the number of polishing passes, it is significantly reduced from 1 × 0 to 2 × 0 passes at the modulation frequencies *f_m_* of 4 and 5 kHz (*f_m_* = 5 kHz: Ra_1 × 0 in SVD_ = 0.72 µm, Ra_2 × 0 in SVD_ = 0.15 µm). Moreover, the achievable roughness Ra increases from 1 × 1 to 2 × 2 passes at *f_m_* = 5 kHz while at *f_m_* = 4 kHz the resulting roughness Ra is stagnating.

**Figure 21:**
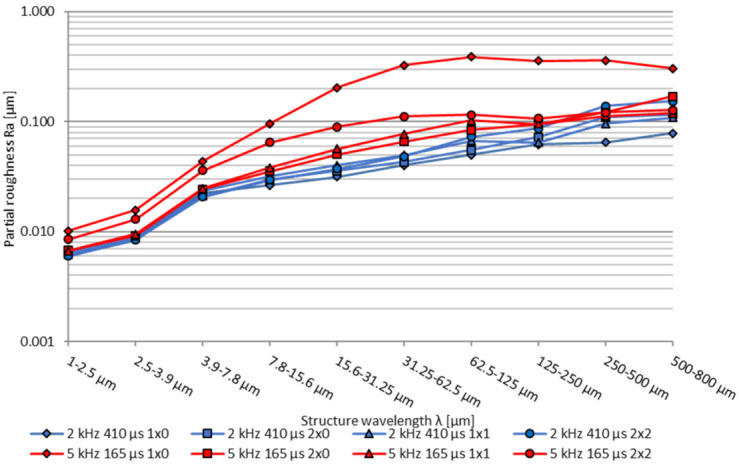
Partial roughness Ra with *f_m_* = 2 kHz, *t_m_* = 410 µs and *f_m_* = 5 kHz, *t_m_* = 165 µs depending on the number of polishing passes, measured in SVD.

Similar to the basic process parameter investigations a Fourier transformation was carried out. Considering the partial roughness Ra, given in Figure 21, the lowest partial roughness was achieved at 1 × 0 polishing passes for a modulation frequency *f_m_* = 2 kHz over the complete wavelength spectrum, see Figure 11. With additional polishing passes especially above a structure wavelength λ of 250 µm a significantly increased roughness can be observed. Possible reasons for that behaviour can be process-introduced waviness caused from increased melt pool dynamics of a deeper melt pool. With a modulation frequency *f_m_* = 5 kHz the lowest partial arithmetic roughness Ra in the range λ = 1–500 µm is achieved with two polishing passes from one direction (2 × 0). Polishing twice (1 × 1) can reach a reduced long wavelength structure above λ = 500 µm, but a slight increased partial roughness in the medium structure wavelength range. Furthermore, it was found that processing at a modulation frequency *f_m_* of 5 kHz with a single pass (1 × 0) resulted in the highest roughness over the complete partial wavelength range. By comparing the modulation frequencies greater differences between the number and direction of polishing passes could be observed at a modulation frequency of *f_m_* = 5 kHz compared to *f_m_* = 2 kHz in the medium- and long wavelength structural range.

#### 3.4.1. Surface Appearance and Topography

**Figure 22:**
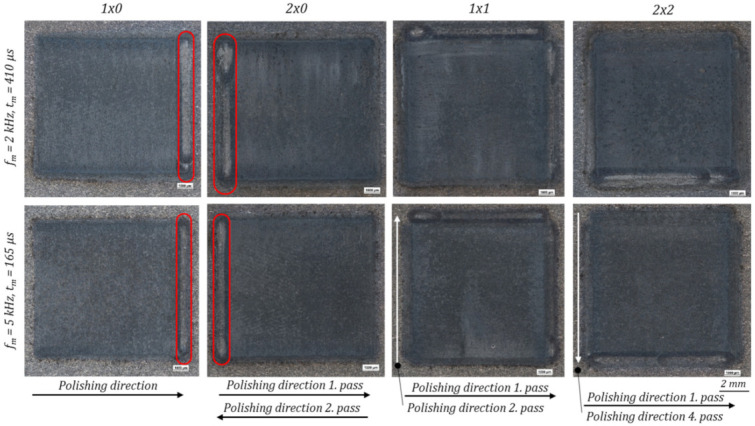
Microscopic images of the laser polished surface with multiple polishing passes (2 × 0, 1 × 1 and 2 × 2) with a 25-fold magnification considering the used modulation frequencies *f_m_* and the modulation time *t_m_*.

Similar to the basic process parameter investigation with one polishing pass, minor differences in the surface appearances can be observed by varying amounts of polishing passes as well as different modulation frequencies *f_m_*, Figure 22. In comparison to single laser polishing, multiple polishing passes leads to a darker surface appearance, which may indicate an increased surface oxidation caused by several re-meltings of the surface layer. Negative effects of the darker layers, which are typically affecting the presence of short wavelength structures cannot be observed in an increased partial roughness with *f_m_* = 2 kHz in the range of λ = 1–7.8 µm (Figure 21). With *f_m_* = 5 kHz and four polishing passes (2 × 2) the partial roughness is increased. Further the melt pool width of the last scanning line is significantly increased, compared to the single laser polishing, which is presumably a consequence of the increased laser beam absorption due to the oxide layers introduced during the first polishing pass. 2 × 0 laser polishing with modulation frequencies *f_m_* of 2 kHz, brighter areas in the upper part of the polishing field are visible, which may indicate smoke caused by impurities or material evaporation due to process overheating. Similar surface effects after pulsed and continuous mode laser polishing on AlSi10Mg revealed an increasing content of oxygen, measured by the use of an EDX [48].

**Figure 23:**
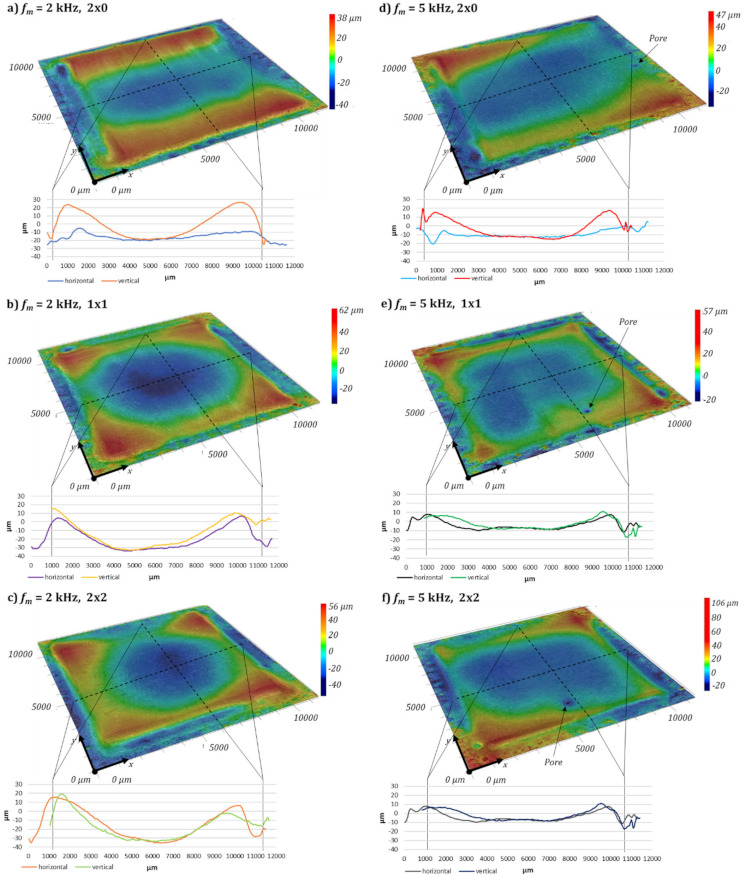
D topography of the laser polished surface in dependency of the number of polishing passes *n* for a modulation frequency of *f_m_* = 2 kHz (**a**–**c**) and *f_m_* = 5 kHz (**d**–**f**) under a 12-fold magnification.

Similar to the process parameter investigation a qualitative analysis of the achieved roughness after laser polishing with multiple polishing passes a 3D topography measurement was carried out, see Figure 23. It should be noted that the height scale differs between each image. In general, multiple laser polishing results in an increased material transport from the centre of the polishing field to the upper and lower borders of the test field, compared to one single polishing pass. When changing the polishing passes from 2 × 0 to 1 × 1 or 2 × 2, the material transport from the centre of the polishing field to the polishing field corners is further increased, see Figure 23a,c. Thereby local immersion of up to −40 µm occur in the centre of the specimen, see Figure 23c. The material displacement is shown at the height profile of the horizontal and vertical lines. Hence, the implemented pump module laser power reduction at the turning points of the scanner pendulum movement is not sufficient for multiple laser polishing passes in order to fully prevent a material relocation.

#### 3.4.2. Residual Surface Structures

**Figure 24:**
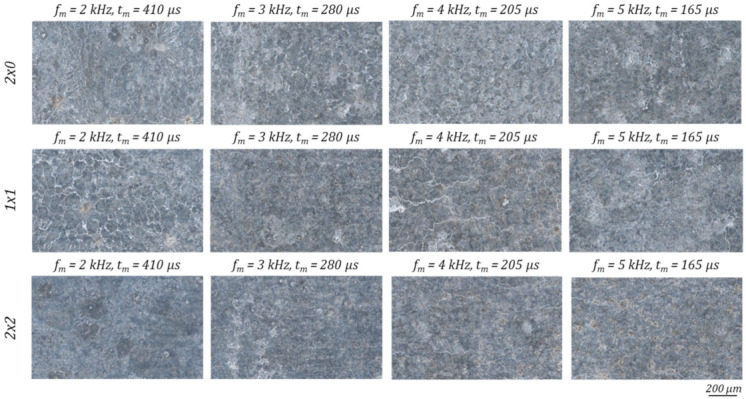
Microscopic differences at a 100-fold magnification of the achieved surface topography after laser polishing with multiple polishing passes.

With exception of four times laser polishing (2 × 2) with *f_m_* = 2 kHz, which exhibits a darker surface appearance, laser polishing with multiple polishing passes results in a similar surface appearance independent from the polishing directions and number of passes, see Figure 24. Especially after four polishing passes the visible bright linear surface structures are no longer present.

**Figure 25:**
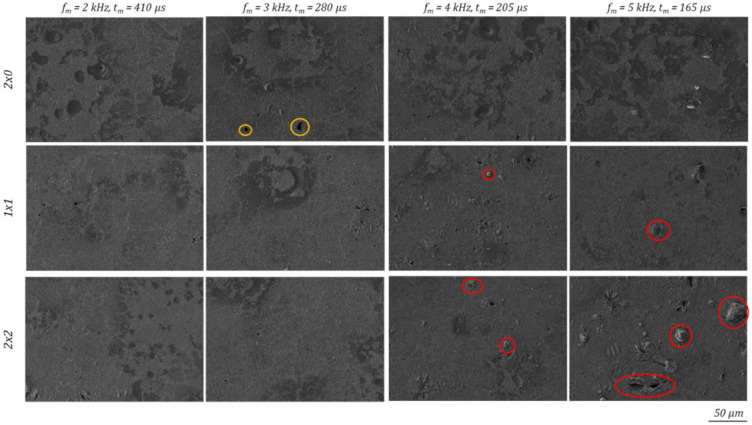
SEM images taken in the centre of the polishing fields at multiple polishing passes under 500-fold magnification.

The micro observation of the polished surface with multiple polishing treatment exhibits mostly a surface without residual structures or defects. Some sporadic surface depressions or pores can be detected at *f_m_* = 3 kHz, 2 × 0 (framed in orange Figure 25). At modulation frequencies *f_m_* above 4 kHz and 1 × 1, or 2 × 2 polishing protruding surface structures are formed (framed in red Figure 25). With *f_m_* = 5 kHz 2 × 2 they reach dimensions of 30 µm and are responsible for the measured increase of the partial arithmetic surface roughness Ra within the structure wavelength range λ = 7.8–62.5 µm, according to Figure 21. 

### 3.5. Roughness Reduction Ability at Varying Initial Surface Roughness

Process parameter investigations of Section 3.2 were carried out with vertical printed L-PBF specimens (fabrication angle α = 90°). However, polishing of 3D parts with complex geometry and fabrication orientations affords a high process stability with regard to strongly varying initial roughness. Specimens with variable fabrication angle between 15 and 90° degree and initial roughness according to Table 2 are used. The polishing is executed with *f_m_* = 3 kHz, *t_m_* = 250 µs and two polishing passes from one direction (2 × 0). 

**Figure 26:**
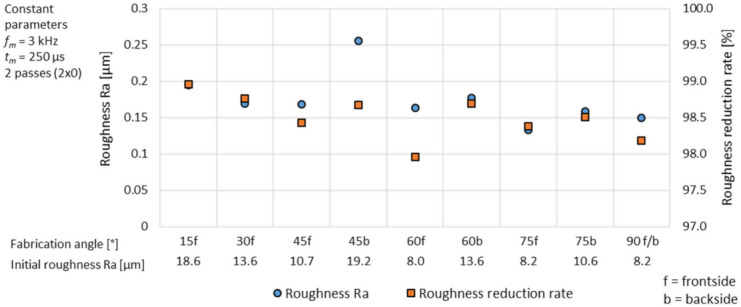
Achievable roughness Ra and relative roughness reduction rate depending on the fabrication angle with *f_m_* = 3 kHz, *t_m_* = 250 µs and two polishing passes (2 × 0).

With rising initial roughness, the achievable surface quality under constant process parameters is reduced, see Figure 26. Overall, the surface roughness variation is in the range of 0.13–0.26 µm. Thus, the achievable surface roughness Ra by laser polishing with modulated laser radiation is directly affected by the initial surface roughness. The process stability and the roughness reduction ability, given by the relative roughness reduction rate, is highly stable between 97.95 and 98.95%, but slightly decrease with a smaller initial roughness. 

**Figure 27:**
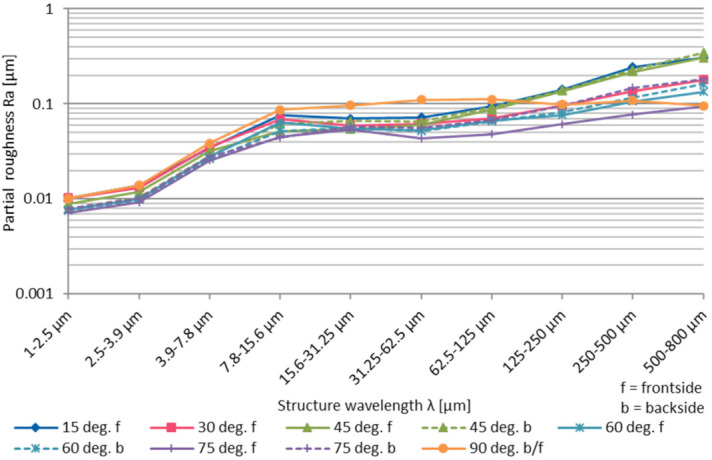
Partial roughness Ra over the structure wavelength λ depending on the fabrication angle α.

The residual partial arithmetic roughness Ra depending on the structure wavelength shows greater differences in the medium to long wavelength range above λ = 62.5 µm, see Figure 27. With decreasing fabrication angle the amount of long periodic residual surface structures increase. While at the structure wavelength range of λ = 31.25–62.5 µm the partial roughness differs between Ra = 0.04 µm–0.11 µm, at λ = 500–800 µm structure wavelength the partial arithmetic roughness Ra is in the range of 0.09–0.35 µm. With 90° fabrication angle at the medium wavelength range from λ = 15.6–125 µm a significant higher roughness can be observed.

### 3.6. Influence of the Modulation Parameters on the Melt Pool Dimension and Thermal Process Stability

**Figure 28:**
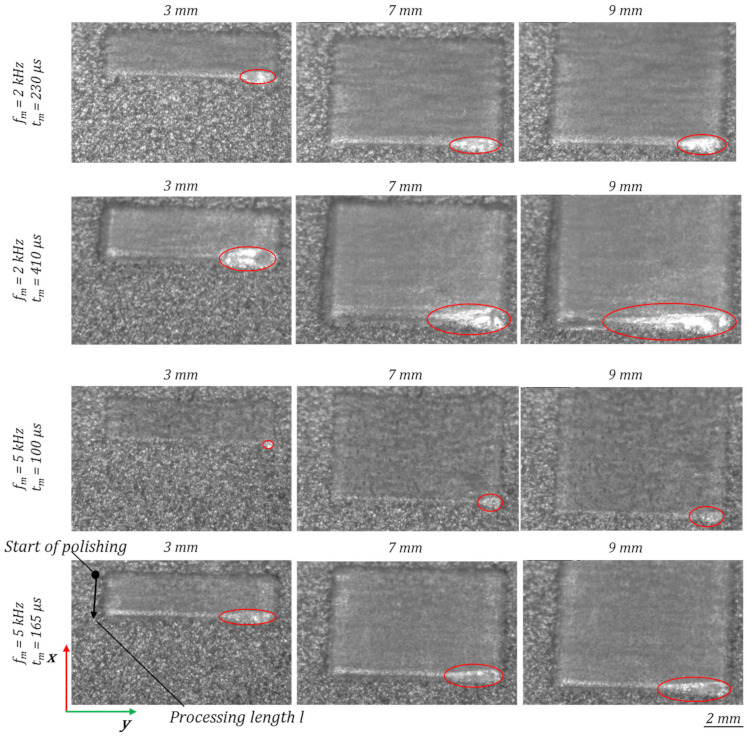
Melt pool dimensions at a processing length of 3 mm, 7 mm and 9 mm, depending on the modulation time *t_m_* and frequency *f_m_*, analysed by single frames from high-speed camera.

The dimension of the melt pool, depending on the process parameters and the processing duration, is analysed by means of high-speed camera videos. The melt pool length in scanning/pendulum direction depending on the process parameters and processing length and the polishing duration, is analysed by taking single frames from the high-speed videos. When observing the melt pool dimension straight before the first turning point of the scanner pendulum movement, a significant rise of the melt pool size with increasing modulation time *t_m_* can be seen, Figure 28. Especially at a modulation frequency of *f_m_* = 5 kHz by increasing the modulation time *t_m_* from 100 µs to 165 µs, the melt pool length behind the ongoing laser spot at a processing length of 3 mm is almost four times larger. With progressing polishing process, the size of the melt pool increases steadily.

**Figure 29:**
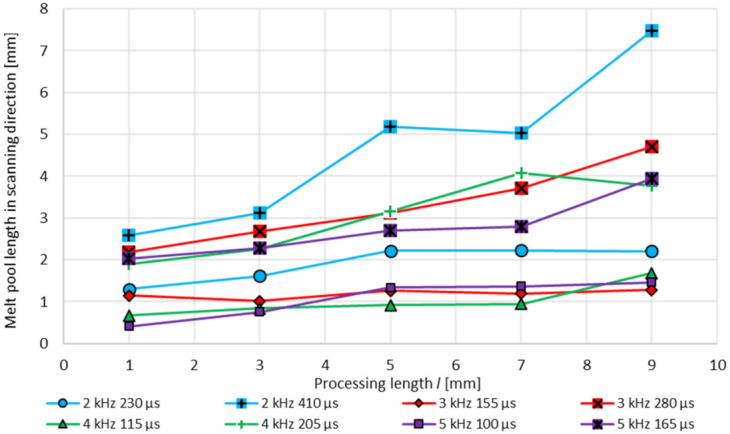
Melt pool length in scanning direction over the processing length *l* depending on the modulation frequency *f_m_* and the modulation time *t_m_*.

The resulting melt pool length in pendulum and in scanning direction after 1 mm processing length varies between 0.4 mm and 2.6 mm, Figure 29. With an ongoing process, laser polishing with modulated laser radiation causes an increase in the melt pool length. Especially with the highest modulation time for each investigated modulation frequency a strong increase occurs, e.g., at *f_m_* = 2 kHz, *t_m_* = 410 µs the melt pool length after a processing length of 9 mm is enlarged by a factor of 2.9. Over all parameters after 9 mm processing length the melt pool length is in the range of 1.3–7.5 mm. 

**Figure 30:**
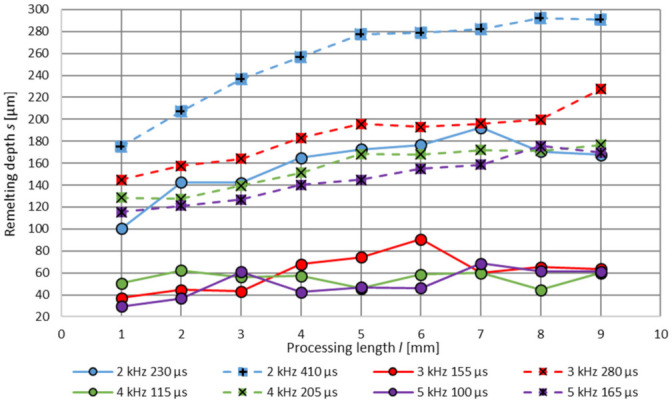
Resulting remelting depth *s* depending on the processing length *l* and process parameter values.

When comparing the increasing melt pool size on the surface over the processing length *l*, observed by the high-speed camera images, with the resulting melting depth *s* over the polishing field length, a correlation can be recognised, see Figure 30. Process parameters with less energy input *ED*, e.g., *f_m_* = 4 kHz, *t_m_* = 115 µs or *f_m_* = 5 kHz, *t_m_* = 100 µs cause an increase in the remelting depth *s* from 50 µm to 61 µm, and from *s* = 29.5 µm to *s* = 61 µm over 8 mm processing length respectively. Process parameters with the high energy input, e.g., 2 kHz, 410 µs lead to a rising melting zone depth from *s* = 175 µm up to *s* = 290 µm at the same processing length. 

Furthermore, an increase in the porosity at high energy densities with rising processing length can be detected, e.g., with *f_m_* = 2 kHz, *t_m_* = 410 µs, Figure 16. 

### 3.7. Comparison to Continuous and Pulsed Laser Operation Modes

The previously shown results revealed that modulated laser polishing with high energy densities can achieve the highest roughness reduction rates, but the process is highly thermal affected by heating up the surrounding material, which results in a steady increasing melt pool and melting depth *s*. In the following, a comparison between continuous (CW) [45], pulsed (PW) [45] and modulated laser operation mode takes place. Thereby, the achievable roughness and area rate, which is affected by the machining strategy and number of passes, are compared. Thereby it has to be noted, that the average initial roughness Ra in fabrication direction amounted 7.87 µm with pulsed and continuous laser radiation [45], while the initial roughness in this study amounts 12.22 µm.

**Figure 31:**
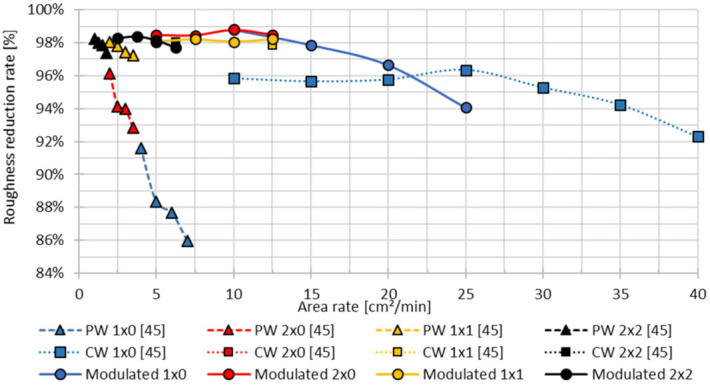
Achieved roughness reduction rate and polishing area rate *AR* with continuous laser radiation (CW) [45], pulsed laser radiation (PW) [45] and modulated laser power.

The roughness reduction rate at pulsed mode laser polishing significantly increases with several polishing passes, see Figure 31. While polishing with one polishing pass can offer a roughness reduction of maximum 91.6% with an area rate *AR* of 4 cm^2^/min, four times laser polishing (2 × 2) achieves the highest roughness reduction of 98.3% at an area rate *AR* of 1 cm^2^/min. Polishing with continuous laser radiation results in a maximum roughness reduction of 96.4% with one polishing pass at an area rate *AR* of 25 cm^2^/min. Multiple polishing offers a further improvement to a maximum roughness reduction of 98.2% with two passings (2 × 0) and *AR* = 12.5 cm^2^/min. In comparison to continuous laser radiation, modulated laser radiation in combination with single laser polishing (1 × 0) offers area rates *AR* between 10 and 20 cm^2^/min and significantly higher roughness reduction rates in the range of 96.7–98.8%. Multiple laser polishing with modulated laser radiation reaches partly a slightly higher roughness reduction rates compared to CW-laser polishing. So modulated polishing twice 1 × 1 at an area rate of 12.5 µm can improve the roughness reduction rate from 97.9% to 98.7%. In contrast to pulsed mode polishing a roughness reduction with modulated laser radiation of 98% is reached with one polishing pass and an area rate of 15 cm^2^/min, while pulsed mode needs two polishing passes (1 × 1) with an area rate *AR* of 2 cm^2^/min. So modulated laser polishing can improve the area rate by a factor of 7.5.

3D printing of complex geometries by L-PBF results in a steady changing orientation of the surface with regard to the fabrication platform. The orientation, given by the fabrication angle, causes major surface roughness variations of the parts between 8.0 and 19.2 µm, according to Table 2. The common pulsed and continuous laser operation modes have shown a direct influence between initial surface and achievable surface roughness by laser polishing [49]. The roughness reduction rate is almost constant. Pulsed mode laser polishing with two polishing passes (2 × 0) and an area rate *AR* of 2 cm^2^/min result in a roughness variation of 0.25–0.5 µm (∆Ra = 0.25 µm), while polishing with continuous radiation with one polishing pass (1 × 0) and an area rate *AR* of 25 cm^2^/min result in a roughness range between 0.29 µm and 0.59 µm, see Table 6. Laser polishing twice (2 × 0) with modulated laser power with a modulation frequency *f_m_* of 3 kHz, a modulation time *t_m_* of 250 µs and an area rate *AR* of 7.5 cm^2^/min offers in comparison to the common laser operation modes over all fabrication angles an additional roughness reduction between 40% and 93%. Further a much higher surface homogeneity of the achievable roughness with a roughness variance of Ra = 0.13–0.26 µm, (∆Ra = 0.13 µm) can be achieved.

## 4. Conclusions

In this work a new approach to laser polishing by modulated laser power is introduced. The method has been successfully tested and applied on surfaces of AlSi10Mg L-PBF parts with varying periodic laser power curves. The influence of the modulated energy input on the resulting roughness reduction had been analysed. The remelting depth, the porosity of the treated surface layers and the residual surface structures and defects are studied. In addition, a further roughness reduction ability with multiple polishing passes depending on the modulation parameters as well as the process stability with regard to varying initial surface roughness and the thermal process stability with regard to component heating effects are investigated. The following correlations and findings can be summarised:

1. Laser polishing of AlSi10Mg L-PBF parts with modulated laser power revealed a decreasing roughness with increasing modulation time and rising energy density *ED*, respectively. The highest roughness reduction rates and the smallest roughness variations are achievable with a modulation frequency *f_m_* of 2 kHz and a modulation time *t_m_* of 410 µs, measured in sample vertical direction. Thereby the initial arithmetic roughness of Ra = 12.22 µm is reduced by a factor of 98.8% to Ra = 0.145 µm with one polishing pass. At similar energy densities, higher modulation frequencies lead to a higher roughness reduction rate.
2. With increasing energy density *ED*, a rising remelting zone depth *s* in the range of 50 µm with *ED* = 20.8 J/mm^2^ to 255 µm with *ED* = 83.2 J/mm^2^ occurs. Increasing melt pools cause a rising relative porosity within the melting zone from 0.3% to 4.6%. Furthermore, with increasing remelting depth the amount of large pores increases strongly and reaches pore sizes up to 7425 µm^2^. Medium and large sized pores at the border of the melting zone, created by the L-PBF process, are partially extended by modulated laser polishing.
3. At the turning points of the scanner pendulum movement modulated laser polishing causes material accumulations, which increase with rising modulation time and energy density *ED* as well with multiple polishing passes.
4. The melt pool expansion in scanning direction, the remelting depth and the relative porosity increase with ongoing processing length and processing duration, respectively. The rate of increase, especially with energy densities *ED* above 34 J/mm^2^, reveals that the process exhibits thermal instability.
5. Flattening of medium and long periodic surface structures requires especially high energy densities and remelting depths. Up to *ED* = 40 J/mm^2^ residual spherical particles adhere to the surface with dimensions of several tens of microns. Further linear surface depressions or defects can be detected. Overall, laser polishing leads to a darkening of the surface, which is most likely caused by surface oxidation in the process.
6. Multiple laser polishing:
  (a) Polishing with two and four passing’s leads to a reduced and almost orientation independent surface roughness.
  (b) With the exception of *f_m_* = 2 kHz, polishing twice from one direction (2 × 0) achieves a further significant roughness improvement and the lowest arithmetic roughness Ra at the respective modulation frequency.
  (c) Modulations frequencies above 3 kHz with crossed polishing passes (1 × 1, 2 × 2) exhibit a rising amount of spherical particles, which results in a rising partial arithmetic roughness Ra in the medium structure wavelengths.
  (d) In comparison to single laser polishing several re-meltings of the surface layer cause a further darkening and increased melt pool width.
7. Modulated laser polishing is highly stable and independent with regards to a varying of the initial surface roughness. Thus, the roughness reduction rate with an initial roughness between 8.0 and 19.2 µm amounts to 97.95–98.95%. Main differences of the achievable surface quality are found in the residual long wavelength structures.
8. Modulated laser polishing offers a higher process efficiency and stability in comparison to pulsed and continuous laser operation modes known from literature. Thus, in relation to pulsed mode polishing roughness reduction rates above 98% are achievable with more than five times higher area rates or in other words the process results are further improved compared to state-of-the-art by a factor of almost 2. In comparison to polishing with continuous polishing with one polishing pass the roughness reduction rate at area rates of 15–20 cm^2^/min is increased, e.g., at 15 cm^2^/min 97.9% vs. 95.7%. Furthermore, the process stability, regarding to varying initial roughness is improved. Thus, the roughness variation after polishing ∆Ra_polished_ is reduced from 0.25 µm with pulsed laser radiation [49], and 0.30 µm with continuous laser radiation [49] to 0.13 µm.

## Figures and Tables

**Table 1:** Initial mean surface roughness Ra and Rz of the vertical printed sample (fabrication angle α = 90°), used for the parameter investigations.

Initial Surface Roughness	Ra _avg_	Ra _min_	Ra _max_	Rz _avg_	Rz _min_	Rz _max_
In sample vertical direction	12.22 µm	9.57 µm	14.62 µm	73.5 µm	64.4 µm	82.9 µm
Perpendicular to the sample vertical direction	11.20 µm	9.54 µm	12.99 µm	68.1 µm	58.5 µm	74.6 µm

**Table 2:** Initial arithmetic surface roughness Ra of the samples depending on the fabrication angle and side after additive manufacturing, measured in SVD.

	Initial Arithmetic Roughness Ra [µm]					
Fabrication angle [°]	15	30	45	60	75	90
Frontside (f)	18.6	13.6	10.7	8.0	8.2	8.2
Backside (b)	-	-	19.2	13.6	10.6	

**Table 3:** Measured laser modulation characteristic with average, minimum and peak laser power depending on the modulation time *t_m_* and modulation frequency *f_m_*.

Modulation Time *t_m_* [µs]	Modulation Frequency *f_m_* [kHz]	Average Laser Power [W]	Minimum Laser Power [W]	Peak Laser Power [W]
230	2	801	60	1680
300		1026	96	1696
350		1188	208	1712
410		1387	528	1712
155	3	811	128	1552
200		1027	256	1648
250		1270	560	1696
280		1420	864	1712
115	4	803	240	1408
140		962	368	1520
180		1223	672	1632
205		1389	960	1680
100	5	865	416	1296
120		1029	560	1424
140		1193	784	1536
165		1393	1168	1568

**Table 4:** Constant process parameters.

Process Parameter	Unit	Value
Pendulum width *x*	mm	10
Field length *l*	mm	10
Process gas flow rate $f_{rate}$	l/min	15
Residual Oxygen $O_{res}$	ppm	40
Focal position *z*	mm	12
Beam diameter $d_{s}$	µm	1298
Peak to Peak overlap *PPO_av_*	%	84.6
Track overlap *TO*	%	93.6

**Table 5:** Experimental plan of the basic process parameter investigations with modulated laser power.

Test Series Number	Modulation Time *t_m_* [µs]	Modulation Frequency *f_m_* [kHz]	Pendulum Frequency *f_p_* [Hz]	Axis Velocity *v_f_* [mm/min]	Energy Density *E* [J/mm^2^]	Area Rate *AR* [cm^2^/min]
1	230	2	20	100	48.1	10
2	300				61.6	
3	350				71.3	
4	410				83.2	
5	155	3	30	150	32.4	15
6	200				41.1	
7	250				50.8	
8	280				56.8	
9	115	4	40	200	24.1	20
10	140				28.9	
11	180				36.7	
12	205				41.7	
13	100	5	50	250	20.8	25
14	120				24.7	
15	140				28.6	
16	165				33.4	

**Table 6:** Achieved roughness Ra at pulsed [49], continuous [49] and modulated laser operation modes depending on the fabrication angle and initial roughness, measured in sample vertical direction (SVD).

Fabrication Angle [°]	Initial Roughness Ra [µm]	Achievable Roughness Ra [µm]		
		Laser Operation Mode		
		Pulsed [49]	Continuous [49]	Modulated
15 f	18.6	0.50	0.47	0.19
30 f	13.6	0.37	0.59	0.17
45 f	10.7	0.35	0.45	0.17
45 b	19.2	0.28	0.55	0.26
60 b	8.0	0.29	0.36	0.16
60 b	13.6	0.33	0.42	0.18
75 f	8.2	0.25	0.35	0.13
75 b	10.6	0.25	0.29	0.16
90 f/b	8.2	0.31	0.36	0.15

## Data Availability

The data, presented in this study, are available on request from the corresponding author.

## References

[1] Kempen K., Thijs L., van Humbeeck J., Kruth J.-P. (2012). Mechanical Properties of AlSi10Mg Produced by Selective Laser Melting. Phys. Procedia.

[2] Mower T.M., Long M.J. (2016). Mechanical behavior of additive manufactured, powder-bed laser-fused materials. Mater. Sci. Eng. A.

[3] Hitzler L., Janousch C., Schanz J., Merkel M., Mack F., Öchsner A. (2016). Non-destructive evaluation of AlSi10Mg prismatic samples generated by selective laser melting: Influence of manufacturing conditions. Mater. Werkst..

[4] van Hooreweder B., Lietaert K., Neirinck B., Lippiatt N., Wevers M. (2017). CoCr F75 scaffolds produced by additive manufacturing: Influence of chemical etching on powder removal and mechanical performance. J. Mech. Behav. Biomed. Mater..

[5] Hassanin H., Elshaer A., Benhadj-Djilali R., Modica F., Fassi I., Gupta K. (2018). Surface Finish Improvement of Additive Manufactured Metal Parts. Micro and Precision Manufacturing.

[6] Lee J.-Y., Nagalingam A.P., Yeo S.H. (2021). A review on the state-of-the-art of surface finishing processes and related ISO/ASTM standards for metal additive manufactured components. Virtual Phys. Prototyp..

[7] Yasa E., Kruth J.-P. (2011). Microstructural investigation of Selective Laser Melting 316L stainless steel parts exposed to laser re-melting. Procedia Eng..

[8] Hitzler L., Janousch C., Schanz J., Merkel M., Heine B., Mack F., Hall W., Öchsner A. (2017). Direction and location dependency of selective laser melted AlSi10Mg specimens. J. Mater. Process. Technol..

[9] Gebhardt A., Hötter J.-S., Ziebura D. (2014). Impact of SLM build parameters on the surface quality. RTejournal-Forum Für Rapid Technol..

[10] Calignano F., Manfredi D., Ambrosio E.P., Iuliano L., Fino P. (2013). Influence of process parameters on surface roughness of aluminum parts produced by DMLS. Int. J. Adv. Manuf. Technol..

[11] Yasa E., Kruth J.-P. (2011). Application of Laser Re-melting on selective laser melting parts. Adv. Prod. Eng. Manag..

[12] Bordatchev E.V., Hafiz A.M.K., Tutunea-Fatan O.R. (2014). Performance of laser polishing in finishing of metallic surfaces. Int. J. Adv. Manuf. Technol..

[13] Ross Ingo Prospects of Laser Polishing for Small and Complexly Shaped Parts: High Speed/High Precision Laser Microfabrication. https://www.swissphotonics.net/libraries.files/epmt_2014_Ross.pdf.

[14] Willenborg E. (2006). Polieren von Werkzeugstählen mit Laserstrahlung.

[15] McDonald M.W., Gora W.S., Stevenson S.G., Weston N.J., Hand D.P. (2020). Practical implementation of laser polishing on additively manufactured metallic components. J. Laser Appl..

[16] Wang W.J., Yung K.C., Choy H.S., Xiao T.Y., Cai Z. (2018). Effects of laser polishing on surface microstructure and corrosion resistance of additive manufactured CoCr alloys. Appl. Surf. Sci..

[17] Yung K.C., Wang W.J., Xiao T.Y., Choy H.S., Mo X.Y., Zhang S.S., Cai Z.X. (2018). Laser polishing of additive manufactured CoCr components for controlling their wettability characteristics. Surf. Coat. Technol..

[18] Gora W.S., Tian Y., Cabo A.P., Ardron M., Maier R.R., Prangnell P., Hand D.P. (2016). Enhancing Surface Finish of Additively Manufactured Titanium and Cobalt Chrome Elements Using Laser Based Finishing. Phys. Procedia.

[19] Richter B., Blanke N., Werner C., Vollertsen F., Pfefferkorn F.E. (2019). Effect of Initial Surface Features on Laser Polishing of Co-Cr-Mo Alloy Made by Powder-Bed Fusion. JOM.

[20] Yung K.C., Xiao T.Y., Choy H.S., Wang W.J., Cai Z.X. (2018). Laser polishing of additive manufactured CoCr alloy components with complex surface geometry. J. Mater. Process. Technol..

[21] Li Y., Zhang Z., Guan Y. (2020). Thermodynamics analysis and rapid solidification of laser polished Inconel 718 by selective laser melting. Appl. Surf. Sci..

[22] Temmler A., Pirch N., Luo J., Schleifenbaum J.H., Häfner C.L. (2020). Numerical and experimental investigation on formation of surface structures in laser remelting for additive-manufactured Inconel 718. Surf. Coat. Technol..

[23] Fang Z., Lu L., Chen L., Guan Y. (2018). Laser Polishing of Additive Manufactured Superalloy. Procedia CIRP.

[24] Kumstel J., Witt G., Wegner A., Sehrt J. (2015). Polieren von SLM-Bauteilen mit kontinuierlicher Laserstrahlung. Neue Entwicklungen in der Additiven Fertigung.

[25] Witkin D.B., Patel D.N., Helvajian H., Steffeney L., Diaz A. (2019). Surface Treatment of Powder-Bed Fusion Additive Manufactured Metals for Improved Fatigue Life. J. Mater. Eng. Perform..

[26] Yung K.C., Zhang S.S., Duan L., Choy H.S., Cai Z.X. (2019). Laser polishing of additive manufactured tool steel components using pulsed or continuous-wave lasers. Int. J. Adv. Manuf. Technol..

[27] Breidenstein B., Brenne F., Wu L., Niendorf T., Denkena B. (2018). Effect of Post-Process Machining on Surface Properties of Additively Manufactured H13 Tool Steel. J. Heat Treat. Mater..

[28] Xiao H., Zhou Y., Liu M., Xu X. (2020). Laser polishing of tool steel using a continuous-wave laser assisted by a steady magnetic field. AIP Adv..

[29] Černašėjus O., Škamat J., Markovič V., Višniakov N., Indrišiūnas S. (2019). Surface Laser Processing of Additive Manufactured 1.2709 Steel Parts: Preliminary Study. Adv. Mater. Sci. Eng..

[30] Li Y.-H., Wang B., Ma C.-P., Fang Z.-H., Chen L.-F., Guan Y.-C., Yang S.-F. (2019). Material Characterization, Thermal Analysis, and Mechanical Performance of a Laser-Polished Ti Alloy Prepared by Selective Laser Melting. Metals.

[31] Liang C., Hu Y., Liu N., Zou X., Wang H., Zhang X., Fu Y., Hu J. (2020). Laser Polishing of Ti6Al4V Fabricated by Selective Laser Melting. Metals.

[32] Tian Y., Góra W.S., Cabo A.P., Parimi L.L., Hand D.P., Tammas-Williams S., Prangnell P.B. (2018). Material interactions in laser polishing powder bed additive manufactured Ti6Al4V components. Addit. Manuf..

[33] Yuan J., Liang L., Lin G. (2019). Study on processing characteristics and mechanisms of thermally assisted laser materials processing. Surf. Coat. Technol..

[34] Ma C.P., Guan Y.C., Zhou W. (2017). Laser polishing of additive manufactured Ti alloys. Opt. Lasers Eng..

[35] Marimuthu S., Triantaphyllou A., Antar M., Wimpenny D., Morton H., Beard M. (2015). Laser polishing of selective laser melted components. Int. J. Mach. Tools Manuf..

[36] Chen L., Richter B., Zhang X., Bertsch K.B., Thoma D.J., Pfefferkorn F.E. (2021). Effect of laser polishing on the microstructure and mechanical properties of stainless steel 316L fabricated by laser powder bed fusion. Mater. Sci. Eng. A.

[37] Bhaduri D., Penchev P., Batal A., Dimov S., Soo S.L., Sten S., Harrysson U., Zhang Z., Dong H. (2017). Laser polishing of 3D printed mesoscale components. Appl. Surf. Sci..

[38] Obeidi M.A., McCarthy E., O’Connell B., Ul Ahad I., Brabazon D. (2019). Laser Polishing of Additive Manufactured 316L Stainless Steel Synthesized by Selective Laser Melting. Materials.

[39] Caggiano A., Teti R., Alfieri V., Caiazzo F. (2021). Automated laser polishing for surface finish enhancement of additive manufactured components for the automotive industry. Prod. Eng..

[40] Ukar E., Lamikiz A., Lacalle L.L.D., Pozo D.D., Liebana F., Sanchez A. (2010). Laser polishing parameter optimisation on selective laser sintered parts. Int. J. Mach. Mach. Mater..

[41] Lamikiz A., Sánchez J.A., López de Lacalle L.N., Arana J.L. (2007). Laser polishing of parts built up by selective laser sintering. Int. J. Mach. Tools Manuf..

[42] Heigl R. (2005). Herstellung von Randschichten auf Aluminiumgusslegierungen mittels Laserstrahlung.

[43] Burzic B., Hofele M., Mürdter S., Riegel H. (2016). Laser polishing of ground aluminum surfaces with high energy continuous wave laser. J. Laser Appl..

[44] Schanz J., Hofele M., Hitzler L., Merkel M., Riegel H., Öchsner A., Altenbach H. (2016). Laser Polishing of Additive Manufactured AlSi10Mg Parts with an Oscillating Laser Beam. Machining, Joining and Modifications of Advanced Materials.

[45] Hofele M., Schanz J., Roth A., Harrison D.K., de Silva A., Riegel H. (2021). Process parameter dependencies of continuous and pulsed laser modes on surface polishing of additive manufactured aluminium AlSi10Mg parts. Mater. Und Werkst..

[46] El Hassanin A., Obeidi M.A., Scherillo F., Brabazon D. (2021). CO_2_ laser polishing of laser-powder bed fusion produced AlSi10Mg parts. Surf. Coat. Technol..

[47] Bhaduri D., Ghara T., Penchev P., Paul S., Pruncu C.I., Dimov S., Morgan D. (2021). Pulsed laser polishing of selective laser melted aluminium alloy parts. Appl. Surf. Sci..

[48] Schanz J., Hofele M., Ruck S., Schubert T., Hitzler L., Schneider G., Merkel M., Riegel H. (2017). Metallurgical investigations of laser remelted additively manufactured AlSi10Mg parts. Mater. Und Werkst..

[49] Hofele M., Roth A., Schanz J., Harrison D.K., de Silva A.K.M., Riegel H. (2021). Laser Polishing of Laser Powder Bed Fusion AlSi10Mg Parts—Influence of Initial Surface Roughness on Achievable Surface Quality. Mater. Sci. Appl..

[50] Temmler A., Dai W., Schmickler T., Küpper M.E., Häfner C.L. (2021). Experimental investigation on surface structuring by laser remelting (WaveShape) on Inconel 718 using varying laser beam diameters and scan speeds. Appl. Surf. Sci..

[51] Temmler A., Pirch N. (2020). Investigation on the mechanism of surface structure formation during laser remelting with modulated laser power on tool steel H11. Appl. Surf. Sci..

